# Optimization of monomethoxy poly(ethylene glycol) grafting on Langerhans islets capsule using response surface method

**DOI:** 10.1186/2194-0517-2-7

**Published:** 2013-03-09

**Authors:** Hamideh Aghajani-Lazarjani, Ebrahim Vasheghani-Farahani, Sameereh Hashemi-Najafabadi, Seyed Abbas Shojaosadati, Saleh Zahediasl, Taki Tiraihi, Fatemeh Atyabi

**Affiliations:** 1 Biotechnology Group, Department of Chemical Engineering, Faculty of Engineering, Tarbiat Modares University, P.O. Box 14115–143, Tehran, 1411713116 Iran; 2 Endocrine Physiology Laboratory, Endocrine Research Centre, Research Institute for Endocrine Sciences, Shahid Beheshti University of Medical Sciences, Tehran, 3197619751 Iran; 3 Department of Anatomy, School of Medical Sciences, Tarbiat Modares University, Tehran, 1411713116 Iran; 4 Faculty of Pharmacy, Tehran University of Medical Sciences, Tehran, 1419733171 Iran

**Keywords:** Response surface methodology, Diabetes, PEGylation, Pancreatic islets, Transplantation

## Abstract

Langerhans islet transplantation is a much less invasive approach compared with the pancreas transplantation to ‘cure’ diabetes. However, destruction of transplanted islets by the immune system is an impediment for a successful treatment. Chemical grafting of monomethoxy poly(ethylene glycol) onto pancreatic islet capsule is a novel approach in islet immunoisolation. The aim of this study was to determine an optimized condition for grafting of monomethoxy poly(ethylene glycol) succinimidyl propionate (mPEG-SPA) on islets capsule. Independent variables such as reaction time, the percentage of longer mPEG in the mixture, and polymer concentration were optimized using a three-factor, three-level Box-Behnken statistical design. The dependent variable was IL-2 (interleukin-2) secretion of lymphocytes co-cultured with PEGylated or uncoated control islets for 7 days co-culturing. A mathematical relationship is obtained which explained the main and quadratic effects and the interaction of factors which affected IL-2 secretion. Response surface methodology predicted the optimized values of reaction time, the percentage of longer mPEG in the mixture, and polymer concentration of 60 min to be 63.7% mPEG_10_ and 22 mg/mL, respectively, for the minimization of the secreted IL-2 as response. Islets which were PEGylated at this condition were transplanted to diabetic rats. The modified islets could survive for 24 days without the aid of any immunosuppressive drugs and it is the longest survival date reported so far. However, free islets (unmodified islets as control) are completely destroyed within 7 days. These results strongly suggest that this new protocol provides an effective clinical means of decreasing transplanted islet immunogenicity.

**Electronic supplementary material:**

The online version of this article (doi:10.1186/2194-0517-2-7) contains supplementary material, which is available to authorized users.

## Introduction

Diabetes mellitus type I, insulin dependent diabetes mellitus (IDDM), is an autoimmune disorder which leads to the destruction of insulin-producing pancreatic islets. Current therapies for IDDM include exogenous insulin therapy and pancreas transplantation. Although daily administration of insulin is the standard protocol, deficient control of blood glucose leads to severe complications, such as heart disease, nephropathy, and hypoglycemia (Hill 2004). Currently, pancreas transplantation is the only available option to ‘cure’ diabetes, but this procedure requires major surgery and lifelong immunosuppression therapy. Langerhans islet transplantation is another approach to cure diabetes which is much less invasive (Lakey et al. 2006). However, immune destruction of transplanted islets is an impediment for a successful procedure (Devos et al. 2005). There are two major approaches to immunoisolate islets: islet encapsulation and surface modification (Lee and Byun 2010). Conjugation of polyethylene glycol (PEG) to the surface of Langerhans islets, islet PEGylation, is another novel approach for immunoprotection of the islets (Panza et al. 2000; Scott and Chen 2004). This strategy was originally applied for surface modification of red blood cells (RBC) in which PEG covalently attaches to the surface amino acids of the RBC, thereby masking the major and minor blood group antigens from hosting antibodies (Neu et al. 2003; Sarvi et al. 2006; Hashemi-Najafabadi et al. 2006; Scott and Murad 1998). Based on such encouraging results, PEGylation method was employed for camouflaging pancreatic islets without affecting their viability and functionality. PEGylation also does not increase islets volume (Panza et al. 2000; Lee et al. 2002; Xie et al. 2005). PEGylation reaction happens when the functional group of the polymer, succinimidyl group of mPEG-succinimidyl propionic acid (mPEG-SPA) in this case, conjugates to the amine groups of the collagen matrix of islets, thereby forms a stable amide bond (Jang et al. 2004).

For completing the coverage of the islets surface by PEG without any adverse effects, the reaction factors should be optimized. Contributing factors that affect preceding the reaction are the molecular weight of PEG, polymer concentration, and reaction time (Roberts et al. 2002). Nowadays, many statistical approaches have been known as useful techniques to optimize the process these variables. Box-Behnken or modified central composite design is an independent and nearly rotatable quadratic design, in which the treatment combinations are at the midpoints of the edges of the process space and at the center (Box et al. 1967). Since 1960, Box-Behnken designs have been very popular with experimenters wishing to estimate a second-order model in three or four factors. This popularity is due to these three-level designs’ simplicity and high efficiency. Among all the response surface methods, this method requires fewer runs in a three-factor experimental design (Chung et al. 2007).

At present study, three-level, three-factor experiments was designed by Box-Behnken to observe the event of parameters influencing on islets PEGylation. The factors that are considered to have an effect on PEGylation are (a) reaction time, (b) the percentage of longer mPEG in the mixture, and (c) polymer concentration. The dependent variable is the amount of IL-2 (interleukin-2) secreted during co-culturing of islets with lymphocytes (*Y*).

## Methods

### Isolation of pancreatic islets and PEGylation

The pancreatic islets were obtained from male Wistar rats (250 to 300 g) as described by Lacy and Kostinovsky (1967). Briefly, 10 mL of Hanks’ balanced salt solution (HBSS) containing 2 mg/mL collaganase type V (Sigma Chemical Co., St Louis, MO, USA) was injected into the pancreas. The swollen pancreas was excised and incubated at 37°C for 15 min. The digestion of pancreas stopped by the addition of 20 mL cold HBSS followed by shaking for 1 min to mechanical disruption. Disrupted pancreas was filtrated through 100-μm mesh to remove other tissues. After washing with HBSS containing fetal bovine serum (FBS), the isolated islets were purified by centrifugation in a discontinuous Histopaque 1.077 (Sigma Chemical Co., St Louis, MO, USA) gradient and then washed with RPMI-1640 culture medium (Sigma Chemical Co., St Louis, MO, USA). Islets were segregated by handpicking and finally cultured overnight in RPMI-1640 medium at 37°C in the humidified atmosphere containing 5% CO_2_ to recover.

Activated mPEG (mPEG-SPA) was prepared and characterized as described by Perry and Kwang (2006). For grafting activated mPEG, purified islets were washed twice with HBSS (pH 7.4) followed immediately by suspending in 10 mL HBSS which contained mPEG-SPA. The suspension was incubated at 37°C in humidified air containing 5% CO_2_. After incubation, the PEGylated islets were washed twice with RPMI and suspended in culture medium for co-culturing with lymphocytes.

### Preparation of splenic lymphocytes

The lymphocyte cells were isolated from the spleen using Ficoll density gradients (Amersham Bioscience, Uppsala, Sweden). The spleen was obtained aseptically from male C57BL/6 mouse. After grinding the spleen in HBSS, the obtained splenocytes were diluted with 3 mL HBSS and layered on 4 mL Ficoll carefully. The cell suspension was centrifuged at 400×*g* for 30 min at 4°C, and then the opaque interface containing lymphocytes were transferred into a conical centrifuge tube. After washing with RPMI, the lymphocytes were isolated. The viability of the isolated splenic lymphocytes was determined by staining with trypan blue (Gibco, Paisley, Scotland).

### Co-culture of islets with lymphocyte

Thirty islets were co-cultured with the 5 × 10^5^ lymphocytes in each well of 96-well plate in 200 μL of RPMI medium containing 10% FBS in 95% O_2_ and 5% CO_2_ for 7 days at 37°C. To determine the release of IL-2 from lymphocytes against the islets, 100 μL of the medium was sampled on the fifth day of culturing and frozen at −70°C for subsequent measurement. The same amount of fresh medium was refilled into each well, so the volume of culture medium was maintained at 200 μL/well. Mouse IL-2 was measured by enzyme-linked immunosorbent assay using commercial kit (E-bioscience, San Diego, CA, USA).

### Box-Behnken statistical design for optimization

A three-factor, three-level Box-Behnken design was used to optimize and evaluate the main effects, interaction effects, and quadratic effects. This design is suitable for exploring quadratic response surface and constructing second-order polynomial models. This cubic design is given by a set of points at the midpoint of each edge of multidimensional cube and a center point replicate (Palamakula et al. 2004). The nonlinear computer-generated quadratic model is given in Equation^1^:

1 $$\begin{array}{l} Y=b_{0}+b_{1}A+b_{2}B+b_{3}C+b_{12}\text{AB}+b_{13}\text{AC}\\ \;+b_{23}\text{BC}+b_{11}A^{2}+b_{22}B^{2}+b_{33}C^{2}, \end{array}$$

where *Y* is the measured response associated with each factor level combination; *b*_0_ is an intercept; *b*_1_ to *b*_33_ are the regression coefficients; *A*, *B*, and *C* are the independent variables. The dependent and independent variables are shown in Table 1. These high, medium, and low levels are selected from the preliminary experiments.

**Table 1:** **Variables and Box-Behnken design and the obtained and predicted results**

Run no.	A	B	C	Experimentally derived IL-2 conc. (pg/mL)	Predicted IL-2 conc. (pg/mL)
	Reaction time (min)	mPEG_10_in mixture (%)	Polymer conc. (mg/mL)		
0^a^	0	0	0	270.47	101.44
1	30	0	16	160.01	159.07
2	30	50	22	145.32	148.88
3	30	100	16	125.48	126.39
4	30	50	10	130.10	126.57
5	45	100	22	103.45	98.98
6	45	100	10	100.65	103.28
7	45	0	22	126.17	123.55
8	45	0	10	103.49	107.96
9^b^	45	50	16	105.32	101.44
10^b^	45	50	16	115.93	106.19
11^b^	45	50	16	100.21	101.44
12^b^	45	50	16	110.73	106.19
13^b^	45	50	16	98.79	101.44
14	60	0	16	93.72	92.81
15	60	50	10	98.60	95.04
16	60	100	16	95.30	96.24
17	60	50	22	80.47	84.00

^a^Refers that the experiment was done with untreated (free) islets; ^b^The center points for calculating experimental error.

After generating the polynomial equations relating the dependent and independent variables presented in Table 1, the process was optimized for the desired response. Optimization was performed to obtain the levels *A*, *B* and, *C*, which minimized *Y*.

### Allotransplantation of PEGylated islets

Inbred male Wistar rats were rendered diabetic with a single intraperitoneal injection of 45 mg/kg of streptozotocin (Sigma) freshly dissolved in citrate buffer (pH 4.5) 3 days before transplantation. Only rats with stable non-fasting blood glucose levels of >350 mg/dL over three continuous measurements were considered diabetic and used for the islet allotransplantation. The blood glucose levels were measured in the tail venous blood using a portable blood glucose meter (Optimum, MediSense, Maiden Head, UK).

For islets transplantation, streptozotocin-induced diabetic recipients were anesthetized by intraperitoneal injection of 90 mg/kg ketamine with 8 mg/kg xylazine. The left kidneys were exposed through a small incision and capsulotomy was performed on the surface of the left kidney, then the unmodified islets or PEGylated islets (1,200 islets/recipient) were injected. After allotransplantation, the glucose levels of non-fasting recipients were measured daily between 10:00 a.m. and 12:00 a.m. Transplantations were considered successful if the blood glucose levels returned to normal level of <120 mg/dL for two consecutive days after islet transplantation, and islet rejection was supposed to have occurred if two consecutive blood glucose levels exceeded 200 mg/dL.

### Statistical analysis

Islet survival data are expressed as median ± SD and analyzed using SPSS v.16.0 statistical software. For comparison of the mean values, independent variable *t* test was used. Significance was determined by one sample *t* test considering the *p* value <0.05.

## Results and discussion

### Box-Behnken statistical design for optimization

The Box-Behnken design for the three factors offers fewer experimental runs as compared with that of central composite models, which require 20 runs (Box et al. 1967). The dependent and independent variables for design-generated experimental runs and the amount of secreted IL-2 for each 18 tests, consisting of 17 runs predicted by software plus a control run, are given in Table 1. Table 2 indicates the analysis of variance of the obtained results. The factors with *p* value <0.05 are significant. From these results, it can be concluded that all experiments resulted in decreasing the IL-2 secretion from lymphocytes which is the result of shielding effect of mPEG-SPA. Jang et al. demonstrated that the lymphocytes co-cultured with PEGylated islets could not affect their viability. Their findings suggested that PEGylation can attenuate the immunogenicity of islets via blocking the recognition of immune cells (Jang et al. 2004). The results in Table 2 indicate that these three factors had a profound effect on the islet masking with mPEG-SPA. At experimental runs of 5, 6, 8, 9, and 13 to 17, the IL-2 secretion decreased 60% more compared to the control run.

**Table 2:** **Analysis of variance table**

Factors	***p*** value	F	***df***	Sum of squares
Reaction time(min)	<0.0001	177.16	1	4647.44
mPEG_10_in mixture (%)	0.0099	16.31	1	427.93
Polymer conc.(mg/mL)	0.1800	2.43	1	63.68
*AB*	0.0168	12.43	1	325.98
*AC*	0.0226	10.60	1	278.06
*A* ^2^	0.0085	17.61	1	461.92
*B* ^2^	0.0741	5.07	1	133.03
Model	0.0010	27.12	9	6402.22
Residual	-	-	5	131.17
Lack of fit	0.2572	3.04	3	107.58

The mathematical relationship in the form of polynomial equation for measured response, *Y*, obtained with statistical package Design Expert (version 7, State Ease Inc., Minneapolis, MN, USA) is in (Equation^2^)

2 $$\begin{array}{l} Y=101.44-24.10A-7.31B+2.82C\\ \;+9.03\text{AB}-8.34\text{AC}+11.19A^{2}+6B^{2} \end{array}$$

This polynomial equation represents the quantitative effect of the process variable (*A*, *B*, and *C*) and their interactions on the response *Y*. The model *F* value is 27.12 and implies that the model is significant.

**Figure 1:**
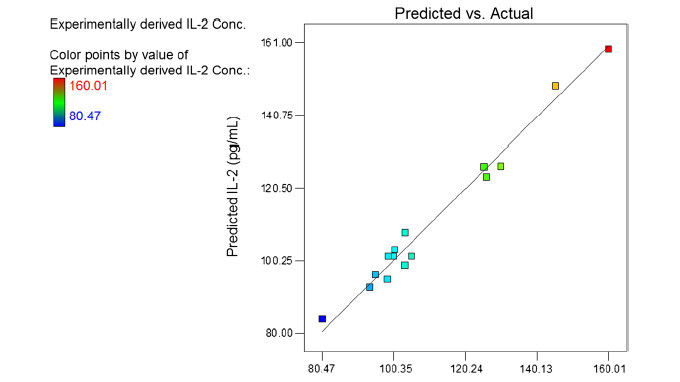
**Parity plot showing the distribution of predicted vs. experimental values of IL-2 secretion.** The bar in the left hand side of the figure indicates the changes from low (blue) to high (red) values.

The values of coefficients *A*, *B*, and *C* are related to the effect of these variables on the response. Coefficients with more than 1 factor term and those with higher order terms represent interaction terms and quadratic relationship, respectively. A positive value represents a favorable effect, while the negative value indicates an adverse effect. In this case *A*, *B*, *AB*, and *AC* have the main effect on the response. The values of *A*, *B*, and *C* were substituted in the equation to obtain the theoretical values of *Y*. In Figure 1, the parity chart, the experimental response values are plotted versus the predicted response values. The points are scattered around the diagonal line which indicate the predicted values and the observed values are in good agreement.

**Figure 2:**
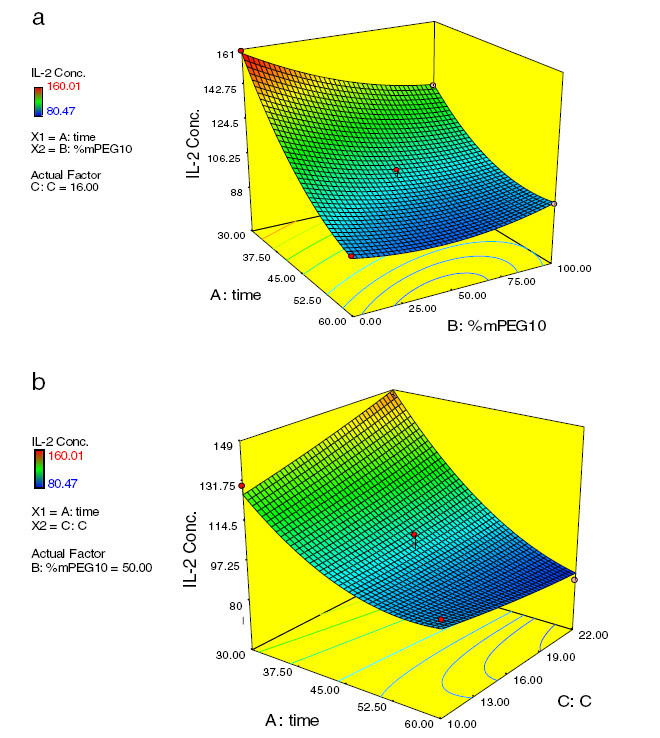
**Response surface and contour plots which show the effect of (a) time and mPEG**_**10**_ **percent and (b) time and polymer concentration on response** ***Y.***

**Figure 3:**
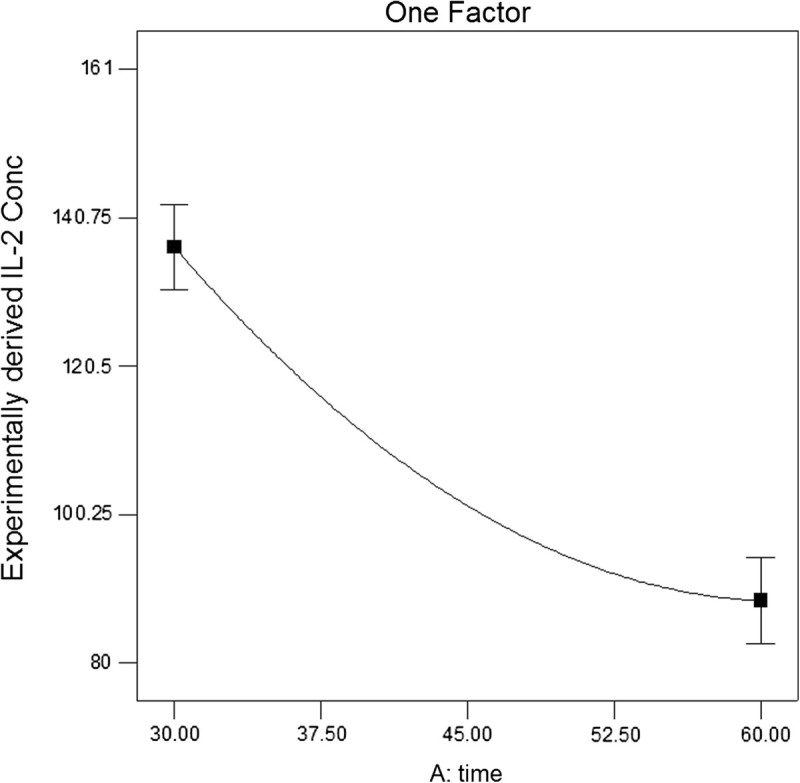
**One-factor plot of reaction time effect on IL-2 secretion.**

Figure 2 is the 2 and 3D views of the main effects of interaction. As it is shown in (a) of Figure 2, IL-2 secretion decreased sharply by increasing time which is completely presented in Figure 3. As shown in (a) of Figure 2, at low reaction times, IL-2 secretion decreased by increasing the percentage of 10 kDa mPEG in the mixture due to the higher shielding effect of long chain polymers on the surface of islets. But at high reaction time, increasing the percentage of 10 kDa mPEG in the mixture of mPEGs (5 and 10 kDa) from 0 to 100 caused an initial decrease in IL-2 secretion with a minimum point occurring at 63.68%. The molecular weight of linear polymers links the chemical and biophysical basis of immunocamouflaging. It is postulated that mPEG with higher molecular weight may be more suitable for surface coating of cells due to its high shielding effect. But Barrou et al. showed that the viscosity of 35 kDa PEG is too high for physiological use. Their results indicated that the optimal chain length at 1.5 mM of PEG is 20 kDa (Neuzillet et al. 2006). The biophysical camouflage of the surface charge is directly proportional to the molecular weight of the grafted polymer. Electrophoretic mobility decreased due to a shift of the shear plane from the surface towards a region of decreased zeta potential. This shift was proportional to the hydrodynamic thickness of the polymer layer and was best achieved with long chain polymers (Lee and Scott 2010). The Flory radii (*R*_F_ root mean square of end to end length of the polymer chain and radius of gyration) of the covalently bound polymers can be calculated by this formula: *R*_F_ = *aN*^(3/5)^ (*a* = number of monomers) (Lee and Scott= 3.5 Å, *N*^2010^). According to this formula, the *R*_F_ (in nm) values of 5 and 10 kDa polymers are 6 and 9.2 nm, respectively. So longer polymers, because of their higher water absorption, higher volume occupation, and movement in larger space, mask the cell surface charges and antigens better, which causes less stimulation of the immune system.

The steric exclusion effect of the grafted polymer chains primarily prevents protein adsorption (Lee and Scott 2010). This effect maximized when chains are grafted at higher density, i.e., with small separation between chains. But the high-density grafting is difficult to achieve with polymers having a large gyration radius (Lee and Scott 2010). When long and short polymers are attached onto surface together, higher density can be achieved. Therefore, this effect can explain why the minimum point occurred at 63.68%. But it should be mentioned that a 5-kDamPEG derivative will have twice the number of reactive groups as a 10-kDa mPEG derivative at the same mass concentration.

The interaction of reaction time and polymer concentration on the immunological response of PEGylated islets is shown in Figure 2b. When polymer concentration increased, the IL-2 secretion decreased in slight incline which is presented in the figure. In our previous work, the surface of the Langerhans islets was coated by cyanuric chloride-activated methoxy(polyethylene glycol). The effect of polymer coating, at two different reaction times and polymer concentrations, was investigated. When polymer concentration and reaction time increased to 40 mg/mL and 90 min, respectively; the immunological response to PEGylated islets decreased to 76.5%, compared to untreated islets (Hashemi-Najafabadi et al. 2007). Recently, we reported that by increasing mPEG-succinimidyl carbonate (5 kDa) concentration up to 22 mg/mL, islets were protected from immune cells more efficiently without affecting their viability and functionality (Aghajani-Lazarjani et al. 2010).

According to the correlation given by Equation^2^, the minimum IL-2 secretion is 83.5622 pg/mL which is expected to be achieved when reaction time, the percentage of mPEG10 in the mixture, and polymer concentration are 60 min, 63.68%, and 22 mg/mL, respectively. The validation experiments were carried out under optimized conditions, and IL-2 secretion was 80.51 ± 6.34 pg/mL. It is in good agreement with the statistically predicted value and confirms the model’s authenticity.

Lee et al. (2002) reported that the optimized concentration of mPEG-SPA (5kDa) and reaction time were 0.25% *w*/*v* and 1 h, respectively. They used one-factor-at-a-time method to optimize the reaction condition, so the effect of the factor interactions was not investigated.

### Transplantation of islets

**Figure 4:**
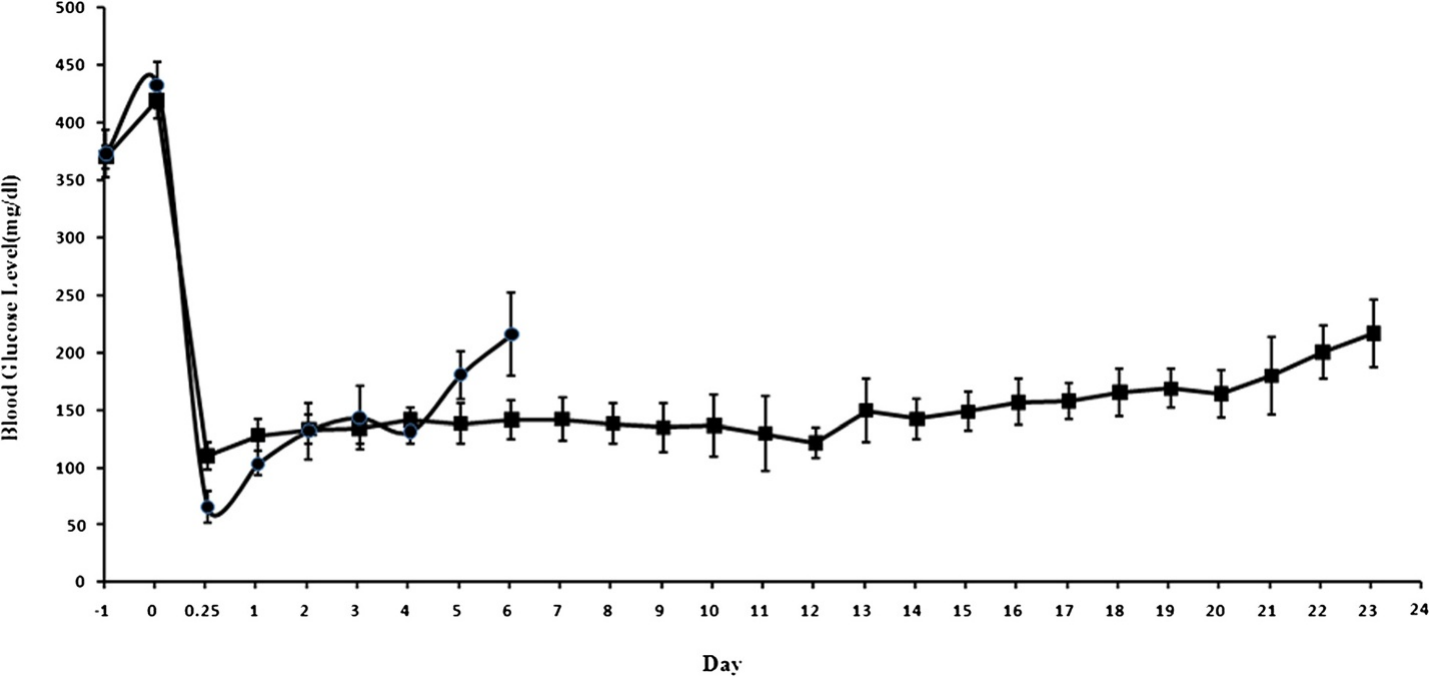
**Changes in non-fasting blood glucose levels of streptozotocin-induced diabetic rat.** Changes in non-fasting blood glucose levels of streptozotocin-induced diabetic rat after transplantation of unmodified islets as control (black circles) and PEGylated islets (black squres). Graft failure was defined as two consecutive plasma glucose concentrations ≥200 mg/dL.

To investigate the effect of PEGylation on islet survival, PEGylated islets obtained at the optimum condition, and unmodified islets (control) were transplanted under the capsule of left kidney. Figure 4 shows the changes in non-fasting blood glucose levels of recipients after transplantation. Six hours after transplantation, the first measurement of blood glucose was taken, and sudden decrease was observed in the case of unmodified islets. The same observation was reported by Teramura and Iwata (2009). These results indicate that the islets were damaged and large amounts of insulin released into the blood, so blood glucose decreased transiently.

Unmodified islets survived for 6.3 ± 1.52 days (mean ± SD; *n =* 3). In contrast, normal glycemia was achieved on transplantation of PEGylated islets for 24.0 ± 3.7 days (mean ± SD; *n* = 3).

Lee et al. reported that the PEGylated islets survive for 12 ± 2.6 days. In their research, 12.5 mg/mL mPEG-SPA (5 kDa) was added to the islets solution. The incubation lasted for 1 h at 37°C in a humidified 5% CO_2_ atmosphere (Jang et al. 2004), which means that the one-layer PEG is insufficient as immunocamouflage. In another study, they showed triple PEGylations method for islet PEGylation. Although they got long survival time for three recipients, more than 100 days, the median survival time was only 19.0 ± 45.6 days (Lee et al. 2007).

## Conclusions

mPEG-SPA could attach covalently on the surface of Langerhans islets without volume increase and cytotoxicity, but the *in vivo* survival time of PEGylated islets is not long enough. So, recent researches’ tendency is to lengthen it. Different approaches have been studied so far such as cyclosporine administration and triple PEGylations method. The aim of this study was to prolong PEGylated islets survival time; therefore, the optimization of PEGylation reaction was studied. The variables of PEGylation reaction were optimized using a response surface method, the Box-Behnken design. The reaction variables such as reaction time, percentage of mPEG_10_ in the mixture, and polymer concentration showed a significant effect on IL-2 secretion from lymphocytes co-cultured with PEGylated islets. The optimum value of these was found to be 60 min, 63.7%, and 22 mg/mL, respectively. The optimized condition was applied for PEGylation, and PEGylated islets were transplanted under the kidney capsule of diabetic rats. The *in vivo* survival time of PEGylated islets increased to 24.0 ± 3.7 days without using immunosuppressive drugs. Thus, single PEGylation at optimum condition intensified islets camouflaging.

## Authors’ original submitted files for images

Below are the links to the authors’ original submitted files for images. Authors’ original file for figure 1 Authors’ original file for figure 2 Authors’ original file for figure 3 Authors’ original file for figure 4 Authors’ original file for figure 5 Authors’ original file for figure 6

## Abbreviations

FBS: Fetal bovine serum
HBSS: Hanks’ balanced salt solution
IDDM: Insulin dependent diabetes mellitus
IL-2: Interleukin 2
mPEG-SPA: Monomethoxypoly(ethylene glycol) succinimidyl propionate
PEG: Polyethylene glycol
RBC: Red blood cells.
