# Supplementary figures and images for: Optimization of monomethoxy poly(ethylene glycol) grafting on Langerhans islets capsule using response surface method
Source: Prog Biomater. 2013 Mar 9;2:7. doi: 10.1186/2194-0517-2-7 (PMC5151098; doi:10.1186/2194-0517-2-7)

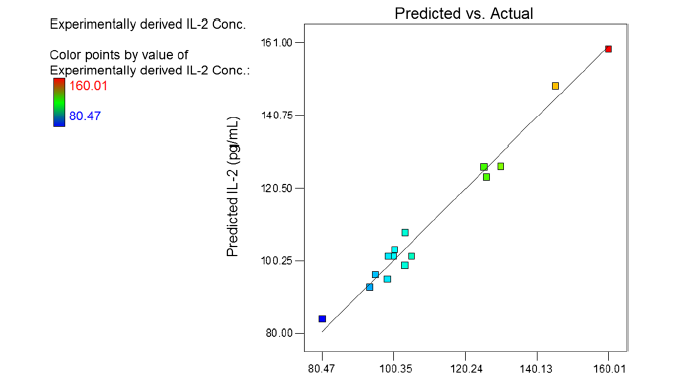

Supplement: Supplementary file 1 — Authors’ original file for figure 1 [file 40204_2012_12_MOESM1_ESM.png]

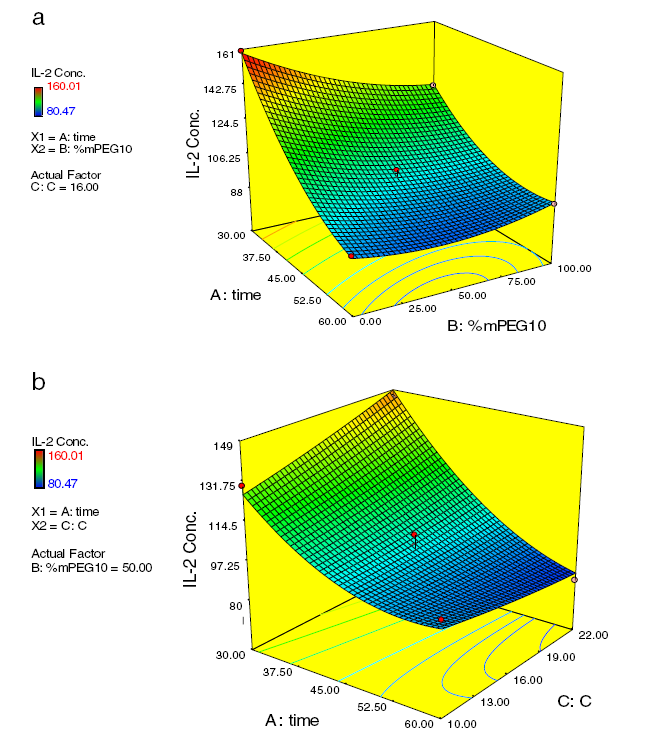

Supplement: Supplementary file 2 — Authors’ original file for figure 2 [file 40204_2012_12_MOESM2_ESM.png]

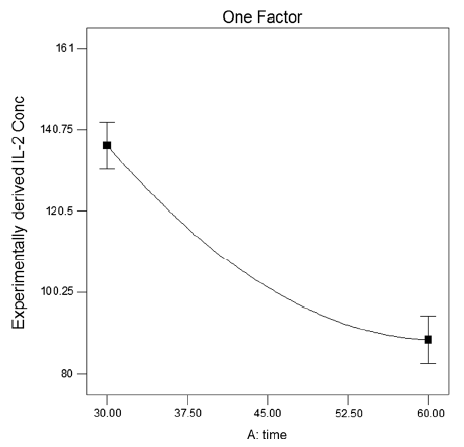

Supplement: Supplementary file 3 — Authors’ original file for figure 3 [file 40204_2012_12_MOESM3_ESM.png]

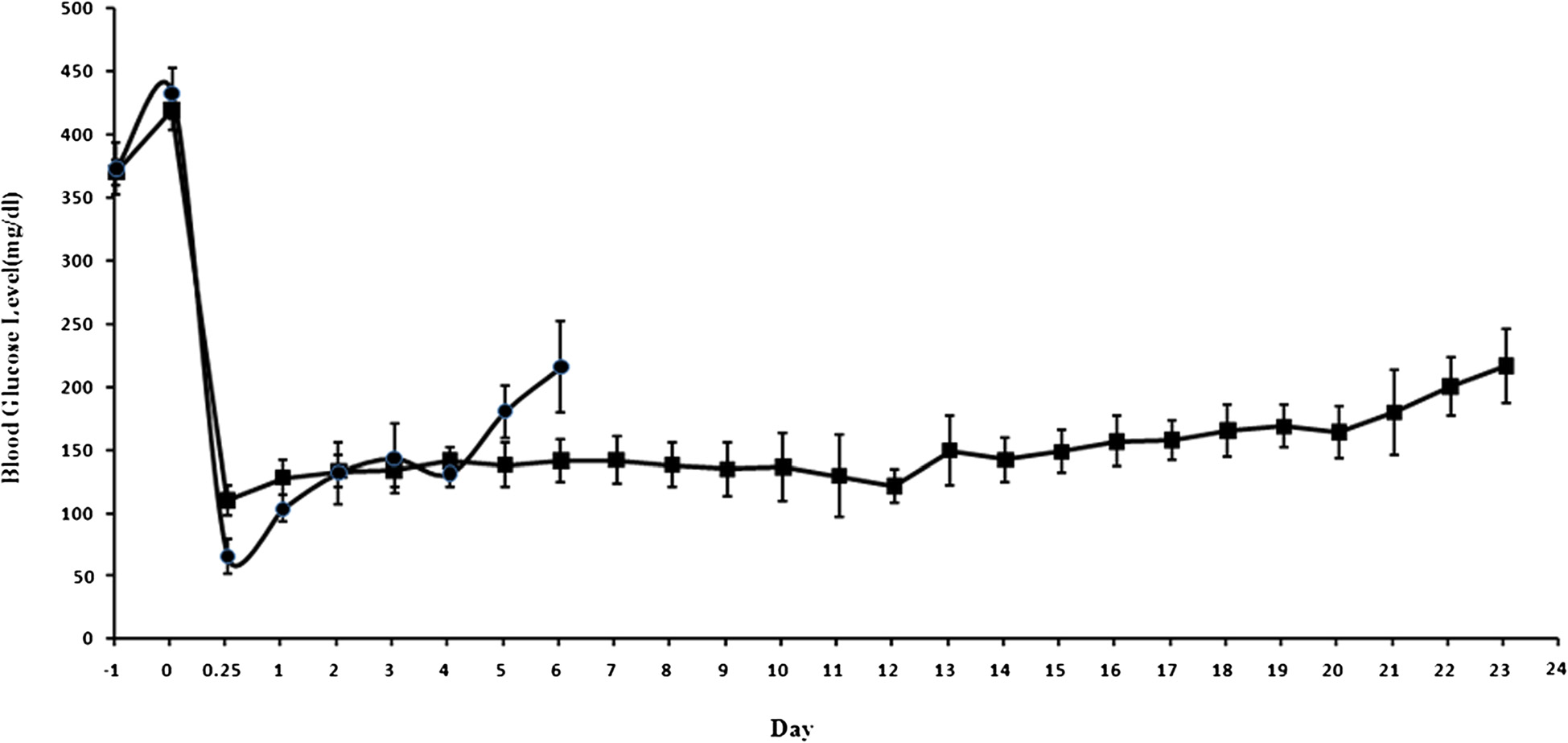

Supplement: Supplementary file 4 — Authors’ original file for figure 4 [file 40204_2012_12_MOESM4_ESM.bmp]

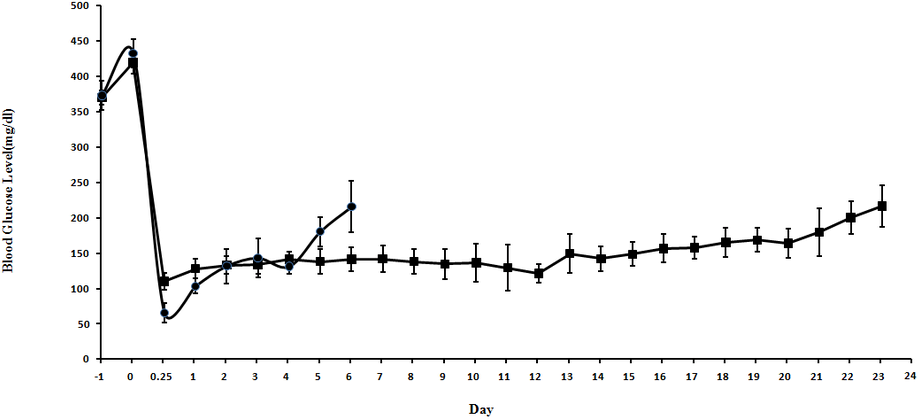

Supplement: Supplementary file 5 — Authors’ original file for figure 5 [file 40204_2012_12_MOESM5_ESM.tiff]

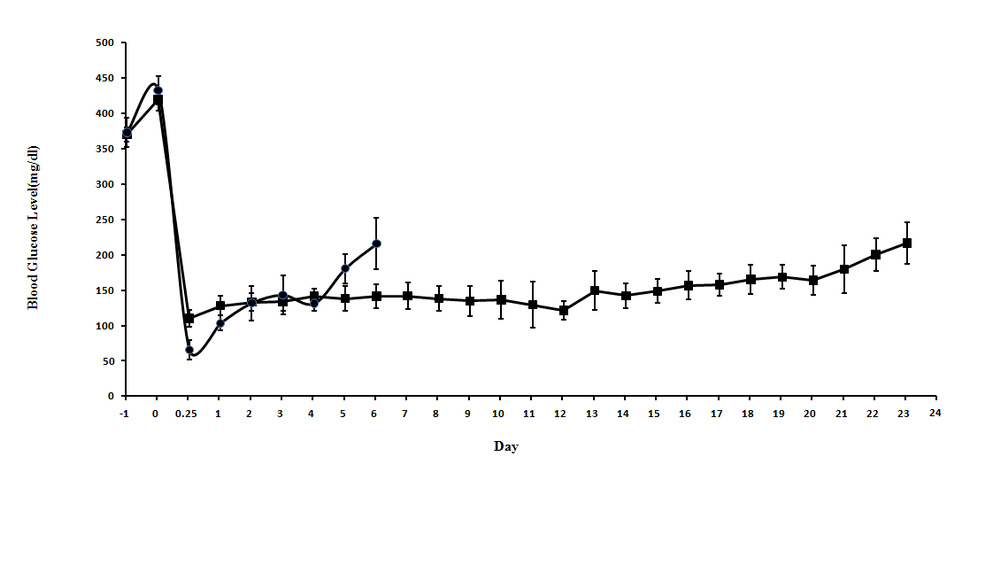

Supplement: Supplementary file 6 — Authors’ original file for figure 6 [file 40204_2012_12_MOESM6_ESM.tiff]
